# Supplementary material for: Adult Diabetes and Prediabetes Prevalence in Kuwait: Data from the Cross-Sectional Kuwait Diabetes Epidemiology Program
Source: J Clin Med. 2020 Oct 25;9(11):3420. doi: 10.3390/jcm9113420 (PMC7694112; doi:10.3390/jcm9113420)
Supplement: Supplementary file 1 [file jcm-09-03420-s001.pdf]

Journal of
Clinical Medicine

MDPI

Supplemental Table S1: Adjusted prevalence of prediabetes by nationality. Adults in Kuwait aged 20 years or more.

	Kuwaitis		Non-Kuwaitis		
	n	Prevalence, % [95% CI]	n	Prevalence, % [95% CI]	*p*
**All**	1684	11.1 [9.7–12.7]	3253	14.3 [13.1–15.5]	0.002
**Sex**					
Men	863	12.2 [10.2–14.5]	1910	15.5 [13.9–17.2]	0.02
Women	821	10.1 [8.2–12.4]	1343	12.2 [10.6–14.1]	0.14
**Age**					
20–29	78	7.6 [3.5–15.6]	292	8.9 [6.1–12.7]	0.74
30–44	679	11.6 [9.4–14.3]	1603	14.8 [13.2–16.7]	0.04
45–59	724	16.0 [13.5–18.9]	1165	19.6 [17.4–21.9]	0.52
60+	203	10.8 [7.3–15.9]	193	13.9 [9.7–19.5]	0.34
**Obesity category**					
Normal BMI	173	6.9 [4.0–11.7]	652	10.4 [8.3–13.0]	0.17
Overweight	600	13.7 [11.1–16.6]	1288	14.5 [12.7–16.5]	0.62
Obese	909	10.8 [8.9–13.0]	1310	16.6 [14.7–18.7]	0.0001
**Waist–hip ratio**					
Normal	338	5.0 [3.2–7.9]	893	10.3 [8.5–12.5]	0.004
Elevated	1326	14.1 [12.3–16.1]	2338	16.3 [14.9–17.9]	0.08
**Self-reported hypertension**					
No	1299	11.2 [9.6–13.1]	2501	14.0 [12.7–15.4]	0.017
Yes	380	9.7 [7.1–13.1]	622	15.4 [12.8–18.5]	0.01
**Self-reported dyslipidaemia**					
No	1263	11.6 [9.9–13.4]	2328	13.7 [12.4–15.2]	0.063
Yes	409	8.6 [6.2–11.7]	637	17.8 [15.0–20.9]	<0.0001
**Family history of diabetes**					
No	331	10.9 [8.0–14.7]	1464	13.8 [12.1–15.6]	0.16
Yes	1178	11.3 [9.6–13.2]	1629	15.0 [13.3–16.8]	0.005
**Education level**					
High school or lower	518	11.6 [9.1–14.6]	1611	13.9 [12.3–15.7]	0.18
University	1166	10.9 [9.2–12.8]	1641	14.6 [12.9–16.4]	0.004
**Monthly household income**					
≤1000 KD (≤approx. $3300)	109	5.5 [2.5–11.5]	2114	14.6 [13.1–16.1]	0.008
>1000 KD (>approx. $3300)	1321	11.3 [9.7–13.1]	457	13.6 [10.7–17.0]	0.19

Supplemental Table S2: Factors associated with prediabetes and diabetes in Kuwaiti nationals.

	Prediabetes		Diabetes	
	OR [95% CI]	*p*	OR [95% CI]	*p*
**Sex**				
Women	1.00		1.00	
Men	1.08 [0.76–1.53]	0.67	1.10 [0.80–1.52]	0.54
**Age**				
20–29	1.00		1.00	
30–44	1.41 [0.48–4.16]	0.53	0.99 [0.29–3.40]	0.98
45–59	2.34 [0.79–6.95]	0.13	2.28 [0.67–7.79]	0.19
60+	2.74 [0.83–9.07]	0.098	4.39 [1.21–15.9]	0.024
**Obesity category**				
Normal BMI	1.00		1.00	
Overweight	1.38 [0.69–2.76]	0.36	1.17 [0.62–2.22]	0.63
Obese	1.78 [0.90–3.50]	0.096	1.88 [1.01–3.50]	0.047
**Waist–hip ratio**				
Normal	1.00		1.00	

Elevated	1.94 [1.15–3.29]	0.014	3.63 [2.06–6.42]	<0.001
**Self-reported hypertension**				
No	1.00		1.00	
Yes	0.75 [0.47–1.21]	0.23	3.07 [2.14–4.40]	<0.001
**Self-reported dyslipidaemia**				
No	1.00		1.00	
Yes	0.45 [0.27–0.73]	0.001	3.73 [2.64–5.26]	<0.001
**Family history of diabetes**				
No	1.00		1.00	
Yes	1.06 [0.70–1.60]	0.79	2.21 [1.45–3.37]	<0.001
**Education level**				
University	1.00		1.00	
High school or less	0.67 [0.45–1.02]	0.059	1.93 [1.38–2.70]	<0.001
**Monthly household income**				
>1000 KD (>approx. $3300)	1.00		1.00	
≤1000 KD (≤approx. $3300)	1.09 [0.52–2.31]	0.82	1.27 [0.64–2.52]	0.50

Supplemental Table S3: Factors associated with prediabetes and diabetes in non-Kuwaiti nationals.

	Prediabetes		Diabetes	
	**OR [95% CI]**	*p*	**OR [95% CI]**	*p*
**Sex**				
Women	1.00		1.00	
Men	1.10 [0.86–1.40]	0.44	2.37 [1.85–3.03]	<0.001
**Age**				
20–29	1.00		1.00	
30–44	1.50 [0.90–2.50]	0.12	1.21 [0.66–2.19]	0.54
45–59	1.85 [1.09–3.15]	0.023	3.50 [1.93–6.37]	<0.001
60+	1.87 [0.94–3.72]	0.076	5.11 [2.54–10.3]	<0.001
**Obesity category**				
Normal BMI	1.00		1.00	
Overweight	1.42 [1.00–2.02]	0.049	1.32 [0.93–1.88]	0.12
Obese	1.60 [1.12–2.29]	0.01	1.79 [1.26–2.55]	0.001
**Waist–hip ratio**				
Normal	1.00		1.00	
Elevated	1.13 [0.85–1.50]	0.40	2.20 [1.61–3.00]	<0.001
**Self-reported hypertension**				
No	1.00		1.00	
Yes	0.80 [0.58–1.10]	0.18	1.98 [1.52–2.58]	<0.001
**Self-reported dyslipidaemia**				
No	1.00		1.00	
Yes	1.23 [0.91–1.64]	0.18	2.07 [1.61–2.68]	<0.001
**Family history of diabetes**				
No	1.00		1.00	
Yes	0.90 [0.71–1.13]	0.36	2.30 [1.82–2.90]	<0.001
**Education level**				
University	1.00		1.00	
High school or less	0.83 [0.65–1.05]	0.12	1.35 [1.07–1.70]	0.013
**Monthly household income**				
>1000 KD (>approx. $3300)	1.00		1.00	
≤1000 KD (≤approx. $3300)	0.95 [0.89–1.01]	0.098	0.95 [0.90–1.01]	0.11
